# 597. The Impact of COVID-19 on Outpatient Intravenous Antimicrobial Therapy (OPAT) in Physician Office Infusion Centers (POICs)

**DOI:** 10.1093/ofid/ofab466.795

**Published:** 2021-12-04

**Authors:** Clifford P Martin, Robin H Dretler, Jorge R Bernett, Barry Statner, Thomas K Sleweon, Quyen Luu, Richard C Prokesch, Kent Stock, Claudia P Schroeder, Thomas C Hardin, Lucinda J Van Anglen

**Affiliations:** 1 Southern Arizona Infectious Disease Specialists, PLC, Tucson, Arizona; 2 Infectious Disease Specialists of Atlanta, P.C., Decatur, GA; 3 Infectious Disease Doctors Medical Group, Walnut Creek, CA; 4 Mazur, Statner, Dutta, Nathan, PC, Thousand Oaks, CA; 5 Infectious Disease Specialists, Highland, Indiana; 6 Central Georgia Infectious Diseases, Macon, Georgia; 7 Infectious Disease Associates, Riverdale, GA; 8 Roper St Francis, Charleston, SC; 9 Healix Infusion Therapy, Sugar Land, TX

## Abstract

**Background:**

The coronavirus disease 2019 (COVID-19) pandemic dramatically affected the provision of healthcare in the U.S. with sharp declines in routine and elective healthcare services. Outpatient clinic visits declined nearly 60% in the early pandemic. We investigated how COVID-19 impacted the provision of OPAT at various Infectious Disease (ID) POICs nationwide.

**Methods:**

Patient (pt) records were evaluated from Jan 2019 – July 2019 and compared to Jan 2020 – July 2020. Data collected included new OPAT pts, demographics, infection type, location prior to OPAT and therapy characteristics. Statistical analysis was performed using Chi-square test with p< 0.05 considered statistically significant.

**Results:**

Fourteen POICs reported data with a total of 2410 new OPAT pts in 2019 and 1807 in 2020, representing a decrease of 25%. Table 1 shows the comparison of OPAT characteristics between 2019 and 2020. Mean age and gender were similar, but there was a significantly higher percentage of pts ≥65 years treated in 2020 (43% vs. 36%, p< 0.001). Infection type and location prior to OPAT were consistent between 2019 and 2020. Primary antimicrobial use was comparable with the exception of cefepime, which showed a greater use in 2020 (14% vs. 11%, p=0.006). OPAT management differed significantly from 2019 to 2020 with fewer pts completing therapy as prescribed in 2020 (85.9% vs. 88.3%, p=0.021), driven largely by more early discontinuations and switches to oral therapy. Other reasons for those not completing therapy were also significant and due primarily to transfer of care to other settings, most commonly the home (1.9% vs. 2.9%, p=0.029). Overall length of therapy was comparable.

Table 1. Comparison of OPAT in 2019 (Pre-COVID) and 2020 (Post-COVID)

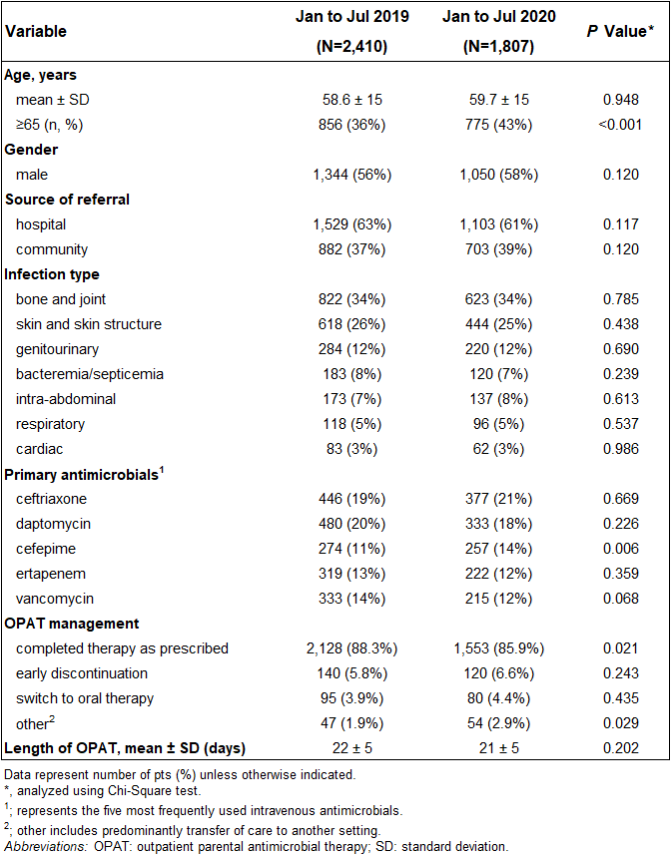

**Conclusion:**

OPAT provided through ID POICs experienced a substantial decrease in pts treated during the first half of 2020 compared to 2019. This was expected with the decline in healthcare services, especially elective procedures. Most pt and treatment characteristics were comparable between years, but interestingly, more elderly received OPAT during the pandemic and fewer completed therapy as planned. Further analysis of these differences can help determine effects of the pandemic on overall health outcomes in the OPAT population.

**Disclosures:**

**Lucinda J. Van Anglen, PharmD**, **Merck & Co.** (Research Grant or Support)

